# ﻿*Chelonopsisguchengensis*, a new species of Lamiaceae from Hubei Province in Central China

**DOI:** 10.3897/phytokeys.253.145834

**Published:** 2025-03-17

**Authors:** Yuhan Xu, Zhengcai Wei, Qiang Fu, Chunlei Xiang, Zhengqun Deng, Houchao Liu, Yuxing Chen, Jun Wen, Xiuqun Liu

**Affiliations:** 1 National Key Laboratory for Germplasm Innovation & Utilization of Horticultural Crops, Huazhong Agricultural University, Wuhan 430070, China Huazhong Agricultural University Wuhan China; 2 Bureau of Natural Resources & Urban and Rural Construction of Xiangyang City, Xiangyang 441003, China Burean of Natural Resources & Urban and Rural Construction of Xiangyang City Xiangyang China; 3 Key Laboratory for Plant Diversity and Biogeography of East Asia, Kunming Institute of Botany, Chinese Academy of Sciences, Kunming 650201, China Kunming Institute of Botany, Chinese Academy of Sciences Kunming China; 4 Administrative Bureau of Nanhe National Nature Reserve of Hubei Province, Gucheng 441700, China Administrative Bureau of Nanhe National Nature Reserve of Hubei Province Gucheng China; 5 Wild Animal and Plant Conservation Station of Xiangyang City, Xiangyang 441003, China Wild Animal and Plant Conservation Station of Xiangyang City Xiangyang China; 6 The Forestry Prospect & Design Institute of Hubei Province, Wuhan 430079, China The Forestry Prospect & Design Institute of Hubei Province Wuhan China; 7 Department of Botany, National Museum of Natural History, MRC166, Smithsonian Institution, Washington, D.C. 20013-7012, USA National Museum of Natural History Washington United States of America

**Keywords:** Central China, Gomphostemmateae, Lamioideae, molecular phylogeny, taxonomy

## Abstract

*Chelonopsis* is a small genus endemic to East Asia. In this study, a new species, *C.guchengensis*, from Nanhe National Nature Reserve, Gucheng County, Hubei Province is described and illustrated. Molecular phylogenetic analyses based on two nuclear ribosomal DNA regions (ETS and ITS) and five plastid DNA markers (*trnL* intron, *trnL*-*trnF*, *trnS*-*trnG*, *psbA*-*trnH*, and *rps16*) were carried out to explore the phylogenetic position of the new species. A close relationship between the new species and *C.giraldii* is supported by molecular phylogenetic and morphological evidence. However, the two species can be easily distinguished from each other by mostly leaf and inflorescence morphology.

## ﻿Introduction

*Chelonopsis* Miq. (Lamiaceae, Lamioideae) is a genus of herbs and shrubs endemic to East Asia (Mabberley 1997). The genus comprises 14 species and three varieties, with most species endemic to China, and southwest China (Xizang, Yunnan and Sichuan) is a center of species diversity for the genus (Li and Hedge 1994; Xiang and Peng 2008; Xiang et al. 2009, 2013; Weckerle et al. 2009). The genus was originally placed within subtribe Melittinae Briq. (Bentham 1848, 1876; Cantino 1985). However, molecular phylogenetic study (Scheen et al. 2010) utilizing chloroplast *rps16* and *trnL-trnF* sequences revealed that it forms a monophyletic group with *Bostrychanthera* Benth. and *Gomphostemma* Wall. ex Benth. Based on this finding, Scheen et al. (2010) established tribe Gomphostemmateae to elucidate the phylogenetic position of these genera, and the tribe was supported by a subsequent study (Bendiksby et al. 2011). Later, a molecular phylogenetic analysis using two nuclear regions and five plastid loci based on comprehensive taxon sampling has confirmed the monophyly of Gomphostemmateae and *Bostrychanthera* was synonymized under *Chelonopsis* (Xiang et al. 2013).

Based on calyx morphology and habit, *Chelonopsis* was divided into two subgenera: subg. Aequidens C. Y. Wu & H. W. Li and subg. Chelonopsis (Wu et al. 1965; Wu 1977). Subgenus Aequidens was further divided into sect. Microphyllum C. Y. Wu & H. W. Li and sect. Aequidens on the basis of leaf blade morphology. Meanwhile, sect. Microphyllum was subdivided into ser. Roseae C. Y. Wu and H. W. Li (with glandular trichomes) and ser. Lichiangenses C. Y. Wu and H. W. Li (without glandular trichomes), a classification system supported by the anatomical studies of the trichomes (Xiang et al. 2010). The infrageneric classification system based on morphology was supported by molecular evidence (Xiang et al. 2013).

During a biological survey of Nanhe National Nature Reserve in Hubei Province, we found a unique species of *Chelonopsis* distributed in Gucheng County. By comparing it with herbarium specimens of *Chelonopsis*, we observed that this species differs from all known species within the genus. We further confirmed its status as a distinct new species based on morphological and molecular phylogenetic evidence, and designate it as *C.guchengensis*.

## ﻿Material and methods

### ﻿Phylogenetic analyses

DNA was extracted from silica-dried leaves using a modified CTAB method (Porebski et al. 1997), and it was then placed at -20 °C for storage. Two nuclear ribosomal regions (ETS, ITS) and five chloroplast regions (*trnL* intron, *trnL-trnF*, *trnS-trnG*, *psbA-trnH*, *rps16*) were amplified by using universal primers, and the sequences of specific primers are shown in Suppl. material 1: table S1 (Taberlet et al. 1991; Wen and Zimmer 1996; Oxelman et al. 1997; Baldwin and Markos 1998; Hamilton 1999; Beardsley and Olmstead 2002). Polymerase chain reaction (PCR) used the following protocol: 2×Super Pfx MasterMix 25 μL; forward primer 2.5 μL (10 μmol/L); reverse primer 2.5 μL (10 μmol/L); template DNA 2 μL (50 ng/μL) and ddH_2_O 18 μL. The program was set as follows: pre-denaturation at 94 °C for 5min, 35 cycles of 30 s at 94 °C, 30 s at 53 °C, 1 min at 72 °C, and a final extension of 7 min at 72 °C (Xiang et al. 2013). After amplification, the PCR products were electrophoresed in a 1% agarose gel stained with GelRed at 200V for 20 min, and then the results were observed under a UV analyzer, and finally the PCR products with the right size and bright bands were used for sequencing (Applied Biosystems 3730xl, Tsingke Biotechnology Co., Ltd.).

To explore the phylogenetic position of the putative new species in *Chelonopsis*, 202 sequences representing 12 genera of Lamiaceae (Xiang et al. 2013) were downloaded from the GenBank (https://www.ncbi.nlm.nih.gov/). *Holmskioldiasanguinea* Retz. was selected as the outgroup. All sequences used in this study together with their accession numbers in GenBank were listed in Suppl. material 1: table S2. All DNA sequences newly obtained were edited and spliced using SeqMan (DNASTAR package. Seqman User Manual. USA: 2020). The edited sequences were concatenated in PhyloSuite v. 3.2.6 (Ronquist et al. 2012) in the order of *trnS-trnG*, *psbA-trnH*, *trnL* intron, *trnL-trnF*, *rps16*, ITS, and ETS, and the sequence alignment was performed in MAFFT v. 7.402 (Katoh and Standley 2013). IQ-Tree v. 2.0.3 (Nguyen et al. 2015) was used to find the optimal model and construct a phylogenetic tree using the maximum likelihood (ML) method (1000 replications) (Felsenstein 1981). The phylogenetic tree was displayed using the online website Interactive Tree Of Life (iTOL) v5 (https://itol.embl.de/) (Letunic and Bork 2021).

### ﻿Morphological observations

Living plants and vouchers of *C.guchengensis* were examined in Yujiagou, Nanhe National Nature Reserve in Hubei Province. The length and width of the leaves were measured using a digital vernier caliper, and a magnifier was used to check the leaf pubescence. A detailed comparison of *C.guchengensis* with other species of the genus was made based on descriptions and herbarium specimens housed in the following herbaria: ANUB, CDBI, CSFI, CSHH, HHBG, HITBC, IBK, IBSC, ISCB, JF, KUN, NWP, LBG, NAS, PE, WUK, XBGH, SYS, and SZG.

### ﻿Distribution and mapping

Collection sites of specimens representing *C.guchengensis* and *C.giraldii* were located with the software of “2bulu” APP V7.8.8 (https://www.2bulu.com/) to determine the known range of each species. Maps were produced in ArcGIS vers. 10.8.2 (Redlands, California, USA) using the free vector and raster map data available through GeoJSON (https://geojson.cn/).

## ﻿Results

### ﻿Sequences and alignment characterization

A total of 272 sequences were used, which included 70 sequences newly generated in this study and 202 sequences downloaded from the GenBank (see Suppl. material 1: table S2). Sequence lengths were 470–523 nucleotides (nt) in *Chelonopsis* (349–510 nt in the other included taxa) for the *trnL* intron, 286–347 nt (185–309 nt in the other included taxa) for the *trnL-trnF* spacer, 470–569 nt (449–572 nt in the other included taxa) for the *trnS-trnG* spacer, 303–361 nt (168–424 nt in the other included taxa) for the *psbA-trnH* spacer, 822–908 nt (802–916 nt in the other included taxa) for the *rps16* intron, 582–653 nt (481–626 nt in the other included taxa) for the ITS1-5.8S-ITS2 region, and 342–483 nt (374–448 nt in the other included taxa) for the ETS region. The resulting combined and aligned sequence matrix contained 4146 positions (including gaps), of which 548 positions belong to the *trnL* intron partition, 375 to the *trnL-trnF* intergenic spacer partition, 457 to the *psbA-trnH* partition, 614 to the *trnS-trnG* partition, 946 to the *rps16* partition, 704 to the ITS partition, and the ETS region contributed 502 bp.

### ﻿Phylogenetic analyses

**Figure 1.**
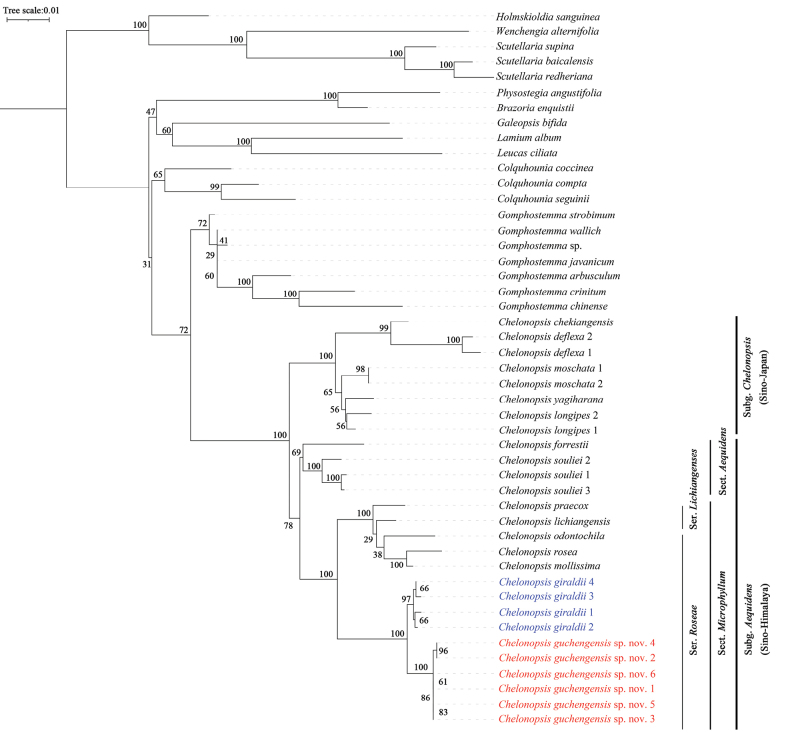
Phylogenetic tree showing the relationship and position of *C.guchengensis* and *C.giraldii* in *Chelonopsis*. Bootstrap values are shown above branches.

As the ML phylogenetic tree (Fig. 1) shows that *C.guchengensis* is a new species in the genus *Chelonopsis*, which could be further placed in subg. Aequidenssect.Microphyllumser.Roseae. Chelonopsisguchengensis and *C.giraldii* formed a subclade with a high support value (SH-aLRT = 100) and is clearly distinguished from other species of the genus.

### ﻿Morphological comparisons

**Figure 2.**
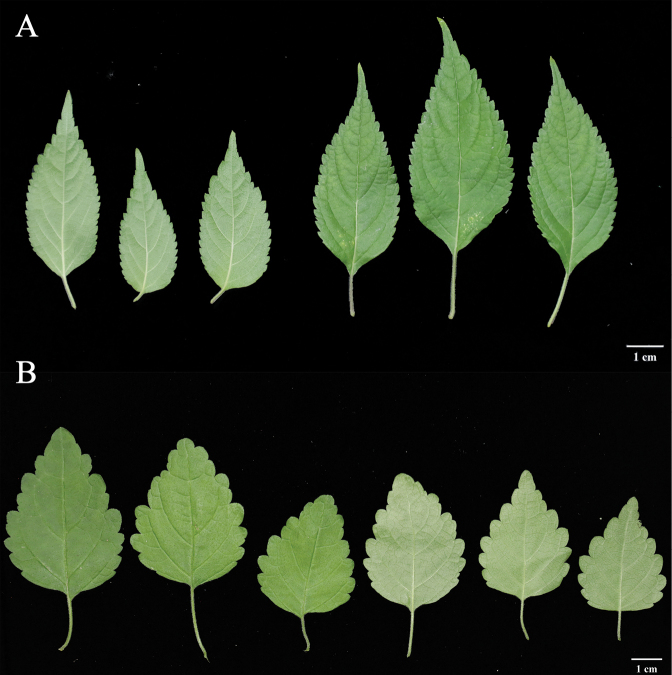
Comparison of *C.guchengensis* and *C.giraldii* in leaf morphology **A***C.guchengensis***B***C.giraldii*.

We compared in detail the morphological differences between *C.guchengensis* and *C.giraldii* because of its close phylogenetic relationships. They showed significant morphological differences (size, shape, lateral veins of leaves) (Table 1, Fig. 2).

**Table 1.** Morphological comparisons of *C.guchengensis* and *C.giraldii*.

Characters	*C.guchengensis*	*C.giraldii*
Life form	semi shrub, high ca.1–2 m	shrubs, high ca.0.3–1.0 m
Size of leaves	ca. 2–10 × 1–5 cm	ca. 2.5–4 × 1.8–2.5 cm
Leaf shape characters	leaf shape oblong-ovoid, base attenuate, margin serrate, apex caudate	leaf shape ovate, base truncate, margin crenate, apex obtuse
Lateral veins	5–7 pairs, impressed above, elevated below	3–4 pairs, impressed above, elevated below
Leaf stalk	ca.1–3 cm, slender, subterete, densely puberulent.	ca.1–2 cm, slender, subterete, densely puberulent.
Stem	terete, striate, much branched, densely white tomentose, greenish or purplish red.	subterete, striate, densely puberulent, much branched, branches slender, leafy.
Inflorescence	cymes axillary, usually 2-flowered.	cymes axillary, 1–3-flowered, usually 1-flowered.
Pedicel	ca.1–2 cm	ca.1–1.4 cm
Bracteole	ca.1–1.5 × 1–2 mm	ca.1 × 1.5 mm
Altitude	570 m	750 m
Distribution	Hubei Province	Shaanxi, Gansu provinces

### ﻿Taxonomy

#### ﻿ Chelonopsis guchengensis

Taxon classificationPlantaeLamialesLamiaceae

X.Q.Liu, Z.C.Wei, Y.H.Xu, Y.X.Chen & J.Wen sp. nov.

8A19CCDC-30BA-5868-B5D7-3D2252FD7090

urn:lsid:ipni.org:names:77358663-1

**Figure 3.**
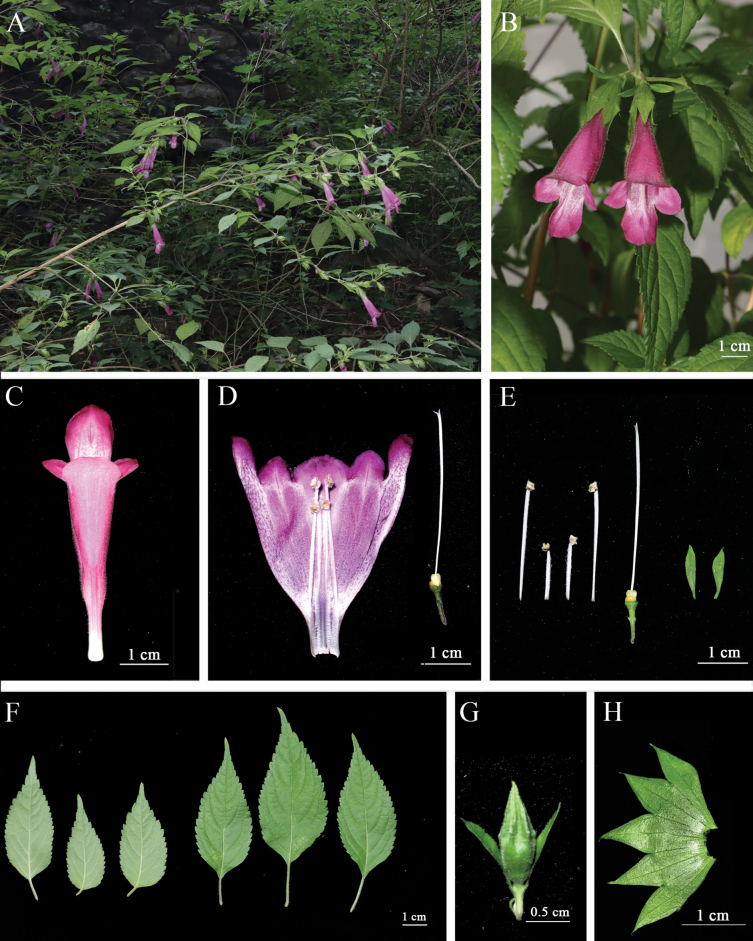
*Chelonopsisguchengensis***A** habit **B** flower **C** front view of flower **D** internal view of corolla **E** pistil, stamens and bracteoles **F** front and back views of the blades **G** flower bud **H** front view of sepal.

Figs 3 , 5, Zhang et al. 2012: fig. 1

##### Type.

China. Hubei • Gucheng County, Nanhe National Nature Reserve, valley nearby Nanhe River, 32°2'8"N, 111°23'48"E, 592 m, 23 October 2023, in ff., *X.Q. LIU 1200* (holotype: CCAU!; isotypes: CCAU!).

##### Diagnosis.

**Figure 4.**
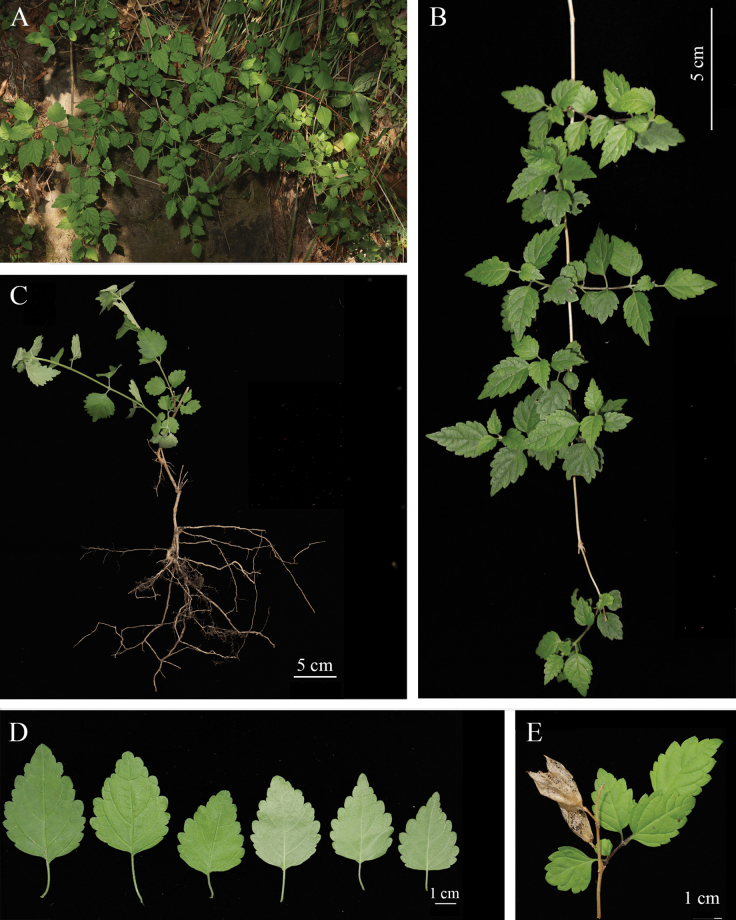
*Chelonopsisgiraldii***A** habit **B** branch **C** plant **D** front and back views of the blades **E** persistent calyxes.

The new species is morphologically similar to *C.giraldii* but differs in having longer and wider leaves (ca. 2–10 cm × 1–5 cm vs ca. 2.5–4 cm × 1.8–2.5 cm), and different leaf shapes (leaf shape oblong-ovoid, base attenuate, margin serrate, apex caudate vs leaf shape ovate, base truncate, margin crenate, apex obtuse in *C.giraldii*) (Fig. 4). The main differences between *C.guchengensis* and other plants in the genus are as follows: leaf shape oblong-ovoid (vs lanceolate in *C.chekiangensis* and ovate in *C.rosea* and *C.mollissima*); usually 2-flowered, pedicel length 1.5 cm (vs 3-flowered or more, pedicel tight and nearly sessile in *C.abbreviata*); bracteoles linear-lanceolate, ca. 1.5 cm long (vs bracteole conspicuous, leaf lanceolate, ca. 2.5 cm long in *C.bracteata*); corolla purplish red (vs yellow in *C.lichiangensis*, *C.odontochila* and milky yellow in *C.souliei*); and calyx apex acuminate (vs gradually aristate in *C.forrestii*).

**Figure 5.**
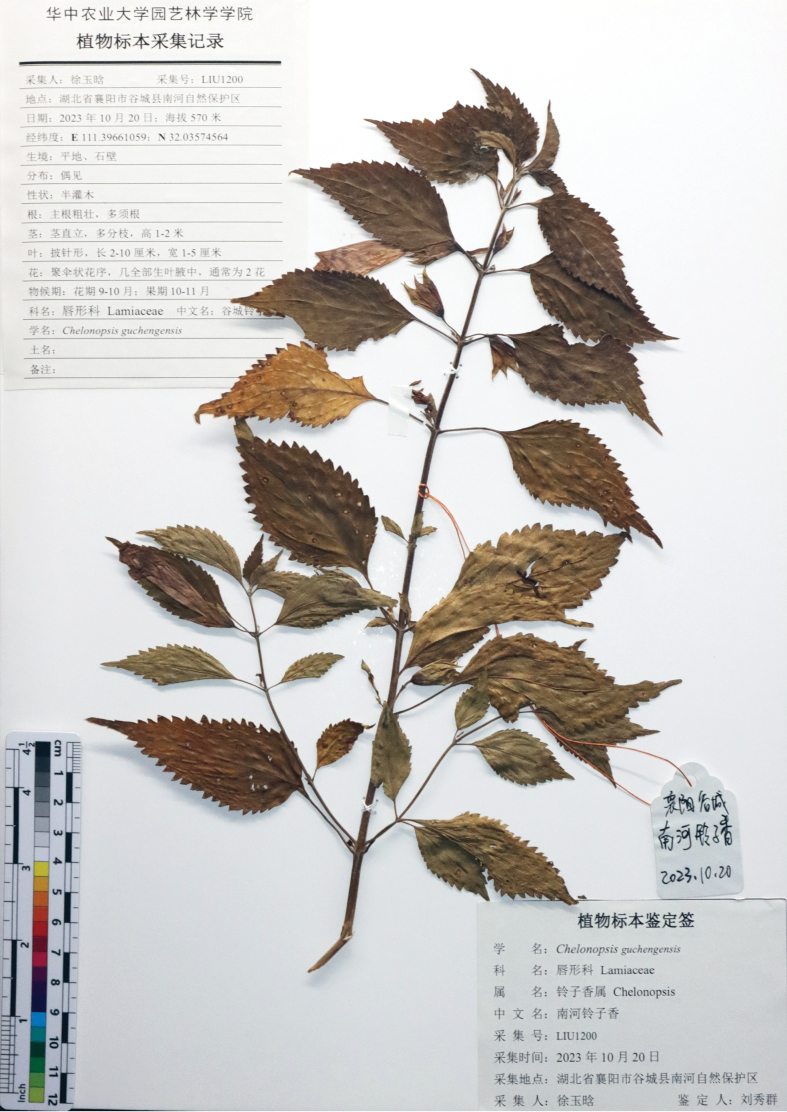
Holotype of *C.guchengensis* sp. nov. X. Q. Liu, Z. C. Wei, Y. H. Xu, Y. X. Chen & J. Wen (*LIU 1200*).

##### Description.

Semi shrubs, ca. 1–2 m tall. ***Stem***: terete, striate, much branched, densely white tomentose, lime green or purple-red. ***Leaves***: ca. 2–10 cm × 1–5 cm, opposite, lanceolate to ovate, apex acuminate, base cuneate, margins doubly serrate, green above, paler below, leaves all cauline, finely punctate, adjacent leaves cruciform, leaf surfaces white bristly, leaf abaxial surfaces glabrous, lateral veins 5–7 pairs, impressed above, elevated below; petiole slender, subterete, ca. 1–3 cm long, densely puberulent. ***Inflorescence***: Cymes axillary, usually 2-flowered. ***Bracteoles***: 2, linear-lanceolate, ca. 1–1.5 cm × 1–2 mm. ***Flowers***: purplish red; calyx with 5 regular, very large, triangular teeth, calyx tube outside white pilose, inside glabrous, apex acuminate, reticulate veins conspicuous, purplish red when young, the same color as the stem, turning green at maturity; corolla ca. 5 cm, upper and lower lips unequal, lower lip obviously longer than upper lip; corolla tube ca. 3 cm, projecting much beyond calyx tube, throat inflated, exterior densely white tomentose, interior glabrous, irregularly spotted along lower lip axis; stamens didynamous, anterior pair longer, filaments filiform, flattened, puberulent, anthers ovoid, whiskered; styles filiform, glabrous, projecting beyond apothecia, apex equally 2-lobed.

##### Phenology.

Flowering from September to October.

##### Distribution and habitat.

**Figure 6.**
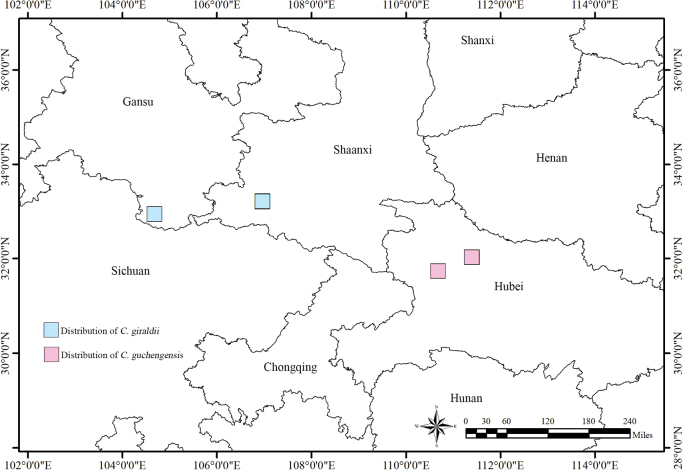
Distribution of *C.guchengensis* and *C.giraldii*. The pink boxes show the distribution areas of *C.guchengensis* and the blue boxes show the distribution areas of *C.giraldii*.

At present, this species is only found in the mountainous area of Nanhe National Nature Reserve, Gucheng County, Hubei Province, China and Shennongjia Forest District, which is commonly found in the valleys near water sources at an altitude of 432–673 m, as well as in moist and fertile thickets (Fig. 6).

##### Etymology.

The new species is named after Gucheng County in Hubei, China where it is distributed. The Chinese name is given as“谷城铃子香”.

## ﻿Discussion

The phylogenetic study placed *Chelonopsisguchengensis* in subg. Aequidenssect.Microphyllumser.Roseae. Within the genus, *Chelonopsisguchengensis* was supported to be sister to *C.giraldii* (Fig. 1). Morphologically, *C.guchengensis* is significantly different from other species in the genus, mainly in its larger leaves and purplish red flowers (Fig. 3). It could be identified by its oblong-ovoid leaf shape, base attenuate, margin serrate, apex caudate and purplish red flowers. Overall, the status of the new species of *C.guchengensis* was clearly established.

The genus *Chelonopsis* was first recorded in Central China (Shennongjia Forest District, Hubei Province) in 2012, and the species was then identified as *C.giraldii* (Zhang et al. 2012). However, the plant from Shennongjia Forest District was clearly different from *C.giraldii.* The leaf shape of the former was oblong-ovoid, base attenuate, margin serrate and apex caudate (fig.1 in Zhang et al. 2012), and that of the latter was ovate, base truncate, margin crenate and apex obtuse. So we identified the plant from Shennongjia Forest District as *C.guchengensis* based on its leaf morphology. As the new species is recorded in Shennongjia Forest District and Gucheng County, Xiangyang City, we speculate that *C.guchengensis* is mainly distributed in the Northwestern part of Hubei Province in Central China. *Chelonopsisgiraldii* is however distributed in Shaanxi and Gansu provinces of Northwestern China.

## Supplementary Material

XML Treatment for Chelonopsis guchengensis

## ﻿Additional information

### Author ORCIDs

Yuhan Xu https://orcid.org/0009-0008-1118-0816

Chunlei Xiang https://orcid.org/0000-0001-8775-6967

Jun Wen https://orcid.org/0000-0001-6353-522X

Xiuqun Liu https://orcid.org/0000-0001-9655-6406

### Data availability

All of the data that support the findings of this study are available in the main text or Supplementary Information.

## Supplementary materials

Supplementary material 1 Supplementary data Data type docx Explanation note **table S1.** Universal primer sequences for amplification of target DNA regions; **table S2.** Species, voucher information, and GenBank accession numbers of the sequences used in this study. Sequence data generated for this study are indicated with an asterisk (*). Missing data are indicated with an n-dash (–). https://binary.pensoft.net/file/1283404This dataset is made available under the Open Database License (http://opendatacommons.org/licenses/odbl/1.0/). The Open Database License (ODbL) is a license agreement intended to allow users to freely share, modify, and use this Dataset while maintaining this same freedom for others, provided that the original source and author(s) are credited.Yuhan Xu, Zhengcai Wei, Qiang Fu, Chunlei Xiang, Zhengqun Deng, Houchao Liu, Yuxing Chen, Jun Wen, Xiuqun Liu

## References

[B1] Baldwin BG Markos S (1998) Phylogenetic utility of the external transcribed spacer (ETS) of 18S-26S rDNA: Congruence of ETS and ITS trees of *Calycadenia* (Compositae). Molecular Phylogenetics and Evolution 10(3): 449–463. 10.1006/mpev.1998.0545 10051397

[B2] Beardsley PM Olmstead RG (2002) Redefining Phrymaceae: The placement of *Mimulus*, tribe Mimuleae, and Phryma. American Journal of Botany 89(7): 1093–1102. 10.3732/ajb.89.7.1093 21665709

[B3] Bendiksby M Thorbek L Scheen AC Lindqvist C Ryding O (2011) An updated phylogeny and classification of Lamiaceae subfamily Lamioideae. Taxon 60(2): 471–484. 10.1002/tax.602015

[B4] Bentham G (1848) Labiatae. In: De CA (Ed.) Pro dromus Systematis Naturalis Regni Vegetabilis.Treuttel and Würtz, Paris, 27–603.

[B5] Bentham G (1876) Labiatae. In: Bentham G Hooker JD (Eds) Genera Plantarum.Reeve, London, 1160–1223.

[B6] Cantino PD (1985) Chromosome studies in subtribe Melittidinae (Labiatae) and systematic implications. Systematic Botany 10(1): 1–6. 10.2307/2418431

[B7] Felsenstein J (1981) Evolutionary trees from DNA sequences: A maximum likelihood approach. Journal of Molecular Evolution 17(6): 368–376. 10.1007/BF01734359 7288891

[B8] Hamilton MB (1999) Four primer pairs for the amplification of chloroplast intergenic regions with intraspecific variation. Molecular Ecology 8: 521–523. 10.1046/j.1365-294X.1999.00510.x 10199016

[B9] Katoh K Standley DM (2013) MAFFT multiple sequence alignment software version 7: Improvements in performance and usability. Molecular Biology and Evolution 30(4): 772–780. 10.1093/molbev/mst010 23329690 PMC3603318

[B10] Letunic I Bork P (2021) Interactive Tree Of Life (iTOL) v5: An online tool for phylogenetic tree display and annotation. Nucleic Acids Research 49(W1): W293–W296. 10.1093/nar/gkab301 PMC8265157 33885785

[B11] Li XW Hedge IC (1994) *Chelonopsis*. In: Wu CY Raven PH (Eds) Flora of China. Science Press, Beijing / Missouri Botanical Garden Press, St.Louis, 17: 135–139.

[B12] Mabberley DJ (1997) A Portable Dictionary of Vascular Plants. Cambridge University Press, Cambridge.

[B13] Nguyen LT Schmidt HA Haeseler A Minh BQ (2015) IQ-TREE: A fast and effective stochastic algorithm for estimating maximum likelihood phylogenies. Molecular Biology and Evolution 32(1): 268–274. 10.1093/molbev/msu300 25371430 PMC4271533

[B14] Oxelman B Liden M Berglund D (1997) Chloroplast rps16 intron phylogeny of the tribe Sileneae (Caryophyllaceae). Plant Systematics and Evolution 206: 393–410. 10.1007/BF00987959

[B15] Porebski S Bailey LG Baum BR (1997) Modification of a CTAB DNA extraction protocol for plants containing high polysaccharide and polyphenol components. Plant Molecular Biology Reporter 15(1): 8–15. 10.1007/BF02772108

[B16] Ronquist F Teslenko M Paul VDM Ayres DL Darling A Höhna S Larget B Liu L Suchard MA Huelsenbeck JP (2012) MrBayes 3.2: Efficient Bayesian phylogenetic inference and model choice across a large model space. Systematic Biology 61(3): 539–542. 10.1093/sysbio/sys029 22357727 PMC3329765

[B17] Scheen AC Bendiksby M Ryding O Mathiesen C Albert VA Lindqvist C (2010) Molecular phylogenetics, character evolution, and suprageneric classification of Lamioideae (Lamiaceae). Annals of the Missouri Botanical Garden 97(2): 191–217. 10.3417/2007174

[B18] Taberlet P Gielly L Patou G Bouvet J (1991) Universal primers for amplification of three non-coding regions of chloroplast DNA. Plant Molecular Biology 17(5): 1105–1109. 10.1007/BF00037152 1932684

[B19] Weckerle CS Huber FK Yang YP (2009) A new hysteranthous species of *Chelonopsis* (Lamiaceae) from southwest China. Novon 19(4): 552–558. 10.3417/2008006

[B20] Wen J Zimmer EA (1996) Phylogeny and biogeography of *Panax* L. (the ginseng genus, Araliaceae): Inferences from ITS of nuclear ribosomal DNA. Molecular Phylogenetics and Evolution 6(2): 167–177. 10.1006/mpev.1996.0069 8899721

[B21] Wu CY (1977) Labiatae. In: Wu CY Li XW (Eds) Flora of China.Science Press, Beijing, 1–649. [In Chinese]

[B22] Wu CY Li XW Xuan SJ Hsuan SJ Huang YC (1965) Materiae ad floram labiatarum sinensium. Acta Phytotaxonomica Sinica 10: 142–146.

[B23] Xiang CL Peng H (2008) Typification of the Name *Chelonopsisalbiflora* (Labiatae). Acta Botanica Yunnanica 30(1): 15–16. 10.3724/SP.J.114310.3724/SP.J.1001.2008.00001

[B24] Xiang CL Liu ZW Xu J (2009) Validation of the name *Chelonopsischekiangensis* (Lamiaceae), a species from eastern China. Novon 19(1): 133–134. 10.3417/2007072

[B25] Xiang CL Dong ZH Peng H Liu ZW (2010) Trichome micromorphology of the east Asiatic genus *Chelonopsis* (Lamiaceae) and its systematic implications. Flora (Jena) 205(7): 434–441. 10.1016/j.flora.2009.12.007

[B26] Xiang CL Zhang Q Scheen AC Cantino PD Funamoto T Peng H (2013) Molecular phylogenetics of *Chelonopsis* (Lamiaceae: Gomphostemma) as inferred from nuclear and plastid DNA and morphology. Taxon 62(2): 375–386. 10.12705/622.11

[B27] Zhang DG Deng T Xu L Cheng GX Zhao YC (2012) *Chelonopsis* Miquel (Lamiaceae), a new record genus for the Floristic Region of Central China. Acta Botanica Boreali-Occidentalia Sinica 32(3): 0619–0621.

