# Supplementary material for: ﻿Chelonopsisguchengensis, a new species of Lamiaceae from Hubei Province in Central China
Source: PhytoKeys. 2025 Mar 17;253:331–42. doi: 10.3897/phytokeys.253.145834 (PMC11933918; doi:10.3897/phytokeys.253.145834)
Supplement: Supplementary material 1 — Supplementary data [file phytokeys-253-331_article-145834__-s001.docx]

Table S1. Universal primer sequences for amplification of target DNA regions.

| Target Genes | Universal Primer Sequences(5' to 3') |
| --- | --- |
| *trnL* intron | primer F:CGAAATCGGTAGACGCTACG |
|  | primer R:GGGGATAGAGGGACTTGAAC |
| *trnL-trnF* | primer F:GGTTCAAGTCCCTCTATCCC |
|  | primer R:ATTTGAACTGGTGACACGAG |
| *trnS-trnG* | primer F:GCCGCTTTAGTCCACTCAGC |
|  | primer R:GAACGAATCACACTTTTACCAC |
| *psbA-trnH* | primer F:CGAAGCTCCATCTACAAATGG |
|  | primer R:ACTGCCTTGATCCACTTGGC |
| *rps*16 | primer F:GTGGTAGAAAGCAACGTGCGACTT |
|  | primer R:TCGGGATCGAACATCAATTGCAAC |
| ETS | primer F:GAGACAAGCATATGACTACTGGCAGGATCAACCAG |
|  | primer R:ATAGAGCGCGTGAGTGGTG |
| ITS | primer F:AGGAGAAGTCGTAACAAG |
|  | primer R:GTTTCTTTTCCTCCGCT |

Table S2. Species, voucher information, and GenBank accession numbers of the sequences used in this study. Sequence data generated for this study are indicated with an asterisk (*). Missing data are indicated with an n-dash (‒).

| **Voucher (herbarium)** | **ETS** | **ITS** | ***psbA-trnH*** | ***rps16* intron** | ***trnL*** | ***trnL-trnF*** | ***trnS-trnG*** |
| --- | --- | --- | --- | --- | --- | --- | --- |
| *Brazoria enquistii* M.W. Turner, Texas, U.S.A., *M.W. Turner 61* (TEX) | ‒ | ‒ | ‒ | HQ911600 | EF546966 | EF546889 | EF546898 |
| *Chelonopsis deflexa* Benth. Guangxi, China, *C.L. Xiang 048* (KUN) | JX893184 | JX893208 | ‒ | JX893258 | JX893282 | JX893311 | JX893340 |
| *Chelonopsis deflexa* Benth. Guizhou, China, *Sino-American Guizhou Bot. Exped. 1923* (A) | ‒ | ‒ | ‒ | FJ854020 | FJ854267 | FJ854154 | ‒ |
| *Chelonopsis chekiangensis* C.Y. Wu, Zhejiang, China, *C.L. Xiang 0602* (KUN) | JX893185 | JX893209 | JX893235 | JX893259 | JX893283 | JX893312 | JX893341 |
| *Chelonopsis forrestii* J. Anthony, Sichuan, China, *C.L. Xiang & H. Peng HP5141* (KUN) | JX893186 | JX893210 | JX893236 | JX893260 | JX893284 | JX893313 | JX893342 |
| *Chelonopsis giraldii* Diels, Shaanxi, China, *C.L. Xiang 025* (KUN) | JX893187 | JX893211 | JX893237 | JX893261 | JX893285 | JX893314 | JX893343 |
| *Chelonopsis lichiangensis* W.W. Sm., Yunnan, China, *C.L. Xiang 020* (KUN) | JX893188 | JX893212 | JX893238 | JX893262 | JX893286 | JX893315 | JX893344 |
| *Chelonopsis longipes* Makino, Japan, *Yamamuro 44619* (KUN) | JX893189 | JX893213 | JX893239 | JX893263 | JX893287 | JX893316 | JX893345 |
| *Chelonopsis longipes* Makino, Japan, *S. Okuyama & N.Maruyana s.n.* (UPS) | ‒ | ‒ | ‒ | FJ854025 | EF546938 | EF54686 | ‒ |
| *Chelonopsis mollissima* C.Y. Wu, Yunnan, China, *C.L. Xiang 05005* (KUN) | JX893190 | JX893214 | JX893240 | JX893264 | JX893288 | JX893317 | JX893346 |
| *Chelonopsis moschata* Miq., Japan, *C.L. Xiang Y0607* (KUN) | JX893191 | JX893215 | JX893241 | JX893265 | JX893289 | JX893318 | JX893347 |
| *Chelonopsis moschata* Miq., cult., *P.D. Cantino 1429* (BHO) | ‒ | ‒ | ‒ | ‒ | FJ854270 | FJ854157 | ‒ |
| *Chelonopsis odontochila* Diels, Yunnan, China, *C.L. Xiang 022* (KUN) | JX893192 | JX893216 | JX893242 | JX893266 | JX893290 | JX893319 | JX893348 |
| *Chelonopsis praecox* Weckerle & F.K. Huber, Sichuan,China, *C.L. Xiang 06070401* (KUN) | JX893193 | JX893217 | JX893243 | JX893267 | JX893291 | JX893320 | JX893349 |
| *Chelonopsis rosea* W.W.Sm., Yunnan, China, *C.L. Xiang 036* (KUN) | JX893194 | JX893218 | JX893244 | JX893268 | JX893292 | JX893321 | JX893350 |
| *Chelonopsis souliei* (Bonati) Merr., Sichuan, China, *C.L. Xiang & H. Peng HP 5240* (KUN) | JX893195 | JX893219 | JX893245 | JX893269 | JX893293 | JX893322 | JX893351 |
| *Chelonopsis souliei* (Bonati) Merr., Tibet, China, *L. Chen & Z.H. Dong CD01* (KUN) | JX893196 | JX893220 | ‒ | JX893270 | JX893294 | JX893323 | JX893352 |
| *Chelonopsis souliei* (Bonati) Merr., Tibet, China, *T. Zhang & al. 08CS651* (KUN) | JX893197 | JX893221 | JX893246 | JX893271 | ‒ | ‒ | JX893353 |
| *Chelonopsis yagiharana* Hisauti & Matsuno, Tokyo, Japan, *C.L. Xiang 0601* (KUN) | JX893198 | JX893222 | JX893247 | JX893272 | JX893295 | JX893324 | JX893354 |
| *Chelonopsis guchengensis* X. Q. Liu, Z. C. Wei, Y. H. Xu, Y. X. Chen & J. Wen, Hubei, China, *X.Q. Liu 1200* (CCAU) | PQ811592* | PQ788474* | PQ815267* | PQ815227* | PQ815237* | PQ815247* | PQ815257* |
| *Chelonopsis guchengensis* X. Q. Liu, Z. C. Wei, Y. H. Xu, Y. X. Chen & J. Wen,, Hubei, China, *X.Q. Liu 1201* (CCAU) | PQ811593* | PQ788475* | PQ815268* | PQ815228* | PQ815238* | PQ815248* | PQ815258* |
| *Chelonopsis guchengensis* X. Q. Liu, Z. C. Wei, Y. H. Xu, Y. X. Chen & J. Wen,, Hubei, China, *X.Q. Liu 1202* (CCAU) | PQ811594* | PQ788476* | PQ815269* | PQ815229* | PQ815239* | PQ815249* | PQ815259* |
| *Chelonopsis guchengensis* X. Q. Liu, Z. C. Wei, Y. H. Xu, Y. X. Chen & J. Wen,, Hubei, China, *X.Q. Liu 1203* (CCAU) | PQ811595* | PQ788477* | PQ815270* | PQ815230* | PQ815240* | PQ815250* | PQ815260* |
| *Chelonopsis guchengensis* X. Q. Liu, Z. C. Wei, Y. H. Xu, Y. X. Chen & J. Wen,, Hubei, China, *X.Q. Liu 1204* (CCAU) | PQ811596* | PQ788478* | PQ815271* | PQ815231* | PQ815241* | PQ815251* | PQ815261* |
| *Chelonopsis guchengensis* X. Q. Liu, Z. C. Wei, Y. H. Xu, Y. X. Chen & J. Wen,, Hubei, China, *X.Q. Liu 1205* (CCAU) | PQ811597* | PQ788479* | PQ815272* | PQ815232* | PQ815242* | PQ815252* | PQ815262* |
| *Chelonopsis giraldii* Diels, Shaanxi, China, *X.Q. Liu 1206* (CCAU) | PQ786000* | PQ614911* | PQ815273* | PQ815233* | PQ815243* | PQ815253* | PQ815263* |
| *Chelonopsis giraldii* Diels, Shaanxi, China, *X.Q. Liu 1207* (CCAU) | PQ786001* | PQ638516* | PQ815274* | PQ815234* | PQ815244* | PQ815254* | PQ815264* |
| *Chelonopsis giraldii* Diels, Shaanxi, China, *X.Q. Liu 1208* (CCAU) | PQ786002* | PQ638517* | PQ815275* | PQ815235* | PQ815245* | PQ815255* | PQ815265* |
| *Chelonopsis giraldii* Diels, Shaanxi, China, *X.Q. Liu 1209* (CCAU) | PQ786003* | PQ638518* | PQ815276* | PQ815236* | PQ815246* | PQ815256* | PQ815266* |
| *Colquhounia coccinea* Wall., Yunnan, China, *C.L. Xiang 046* (KUN) | JX893199 | JX893223 | JX893248 | JX893273 | JX893296 | JX893325 | JX893355 |
| *Colquhounia compta* W.W. Sm., Yunnan, China, *C.L. Xiang HP5134* (KUN), | JX893200 | JX893224 | JX893249 | JX893274 | JX893297 | JX893326 | JX893356 |
| *Colquhounia seguinii* Vaniot, Guizhou, China, *C.L. Xiang 065* (KUN), | JX893201 | JX893225 | JX893250 | JX893275 | JX893298 | JX893327 | JX893357 |
| *Galeopsis bifida* Boenn., Xinjiang, China, *E.D. Liu & C.L. Xiang 086* (KUN) | JX893202 | ‒ | JX893251 | JX893276 | JX893299 | JX893328 | JX893358 |
| *Gomphostemma arbusculum* C.Y. Wu, Yunnan, China, *C.L. Xiang & al. 0161* (KUN) | JX893203 | JX893226 | JX893252 | JX893277 | JX893300 | JX893329 | JX893359 |
| *Gomphostemma chinense* Oliv., Yunnan, China, *C.L. Xiang & H. Peng HP6070* (KUN) | JX893204 | JX893227 | JX893253 | ‒ | JX893301 | JX893330 | JX893360 |
| *Gomphostemma crinitum* Wall. ex Benth., Yunnan, China, *C.L. Xiang & J. Xu 032* (KUN) | JX893205 | JX893228 | JX893254 | JX893278 | JX893302 | JX893331 | JX893361 |
| *Gomphostemma javanicum* (Blume) Benth., *G.E. Juan s.n.* (US) | ‒ | ‒ | ‒ | HQ911595 | HQ911667 | HQ911735 | ‒ |
| *Gomphostemma strobilinum* Benth. var. *acaule* (Kurz ex Hook.f.) Prain, Thailand, *Maxwell 87-1305* (L) | ‒ | ‒ | ‒ | HQ911596 | HQ911669 | ‒ | ‒ |
| *Gomphostemma* sp., Yunnan, China, *C.L. Xiang 03031* (KUN) | ‒ | ‒ | JX893255 | ‒ | JX893303 | JX893332 | ‒ |
| *Gomphostemma wallichii* Prain, Thailand, *M. Tagawa & al. 9498* (US) | ‒ | ‒ | ‒ | ‒ | HQ911668 | HQ911736 | ‒ |
| *Holmskioldia sanguinea* Retz., Hainan, China, *C.L. Xiang 415* (KUN) | ‒ | ‒ | ‒ | ‒ | JX893304 | JX893333 | ‒ |
| *Lamium album* L., Xinjiang, China, E.D. *Liu & C.L. Xiang 128* (KUN) | JX893206 | JX893229 | JX893256 | JX893279 | JX893305 | JX893334 | JX893362 |
| *Leucas ciliata* Benth.,Yunnan, China, *C.L. Xiang 100* (KUN) | JX893207 | JX893230 | JX893257 | JX893280 | JX893306 | JX893335 | JX893363 |
| *Physostegia angustifolia* Fernald, U.S.A., R.A. *Thopson & al. C0655* (GH) | ‒ | ‒ | ‒ | ‒ | EF546941 | EF546865 | EF546906 |
| *Scutellaria baicalensis* Georgi, Shanxi, China, *Q.J. Yuan SXHLW01* (KUN) | ‒ | JX893231 | ‒ | ‒ | JX893307 | JX893336 | ‒ |
| *Scutellaria supina* L., Xinjiang, China, *E.D. Liu &C.L. Xiang 083* (KUN) | ‒ | JX893233 | ‒ | ‒ | JX893308 | JX893337 | ‒ |
| *Scutellaria rehderiana* Diels, Xinjiang, China, *Q.J. Yuan GSWYW01* (KUN) | ‒ | JX893232 | ‒ | ‒ | JX893309 | JX893338 | ‒ |
| *Wenchengia alternifolia* C.Y. Wu & S. Chow, Hainan, China, *C.Y. Wu & S. Chow, B. Li 0151* (IBSC) | ‒ | JX893234 | ‒ | JX893281 | JX893310 | JX893339 | ‒ |
